# Molecular sensitization pattern to house dust mites is formed from the first years of life and includes group 1, 2, Der p 23, Der p 5, Der p 7 and Der p 21 allergens

**DOI:** 10.1186/s12948-022-00182-z

**Published:** 2023-02-03

**Authors:** Serhii Yuriev, Victoria Rodinkova, Vitalii Mokin, Ilona Varchuk, Olena Sharikadze, Yuriy Marushko, Bohdan Halushko, Andrii Kurchenko

**Affiliations:** 1 Medical Centre, DIVERO, Kiev, Ukraine; 2 Department of Clinical Immunology and Allergology, Bohomolets National Medical University, Kiev, Ukraine; 3 Department of Pharmacy, National Pirogov Memorial Medical University, 56, Pirogov Street, Vinnytsia, 21018 Ukraine; 4 Department of System Analysis and Information Technologies, Vinnytsia National Technical University, Vinnytsia, Ukraine; 5 Paediatric Department, Shupyk National Healthcare University, Kiev, Ukraine; 6 Department of Pediatrics of Postgraduate Education, O.O. Bohomolets Medical University, Kiev, Ukraine

**Keywords:** House dust mites, Environmental allergens, Component-resolved molecular diagnostics of allergy, Molecular sensitization pattern, Multiplex allergy test

## Abstract

**Background:**

As the process and nature of developing sensitivity to house dust mites (HDMs) remain not fully studied, our goal was to establish the pattern, nature and timeframe of house dust mite (HDM) sensitization development in patients in Ukraine as well as the period when treatment of such patients would be most effective.

**Methods:**

The data of the multiplex allergy test Alex^2^ was collected from 20,033 patients. To determine age specifics of sensitization, descriptive statistics were used. Bayesian Network analysis was used to build probabilistic patterns of individual sensitization.

**Results:**

Patients from Ukraine were most often sensitized to HDM allergens of group 1 (Der p 1, Der f 1) and group 2 (Der p 2, Der f 2) as well as to Der p 23 (55%). A considerable sensitivity to Der p 5, Der p 7 and Der p 21 allergens was also observed. The overall nature of sensitization to HDM allergens among the population of Ukraine is formed within the first year of life. By this time, there is a pronounced sensitization to HDM allergens of groups 1 and 2 as well as to Der p 23. Significance of sensitization to Der p 5, Der p 7 and Der p 21 allergens grows starting from the age of 3–6. Bayesian Network data analysis indicated the leading role of sensitization to Der p 1 and Der f 2. While developing the sensitivity to group 5 allergens, the leading role may belong to Der p 21 allergen.

**Conclusion:**

The results obtained indicate the importance of determining the sensitization profile using the multi-component approach. A more detailed study of the optimal age for AIT prescription is required as the pattern of sensitization to HDMs is formed during the first year of life.

## Introduction

House dust mites (HDMs) are undoubtedly one of the major respiratory allergens of our times as they are the leading cause of allergic rhinitis [1] and asthma [2], particularly virus-induced [3] asthma. It has been proved that sensitivity to house dust mites may also be the cause of developing atopic dermatitis [4]. It is believed that about 2% of the world population are house dust mite (HDM) sensitive [2]. Thus, in the recent years, HDMs have become a major disease elicitor and a global issue.

It has been established that such mite species as *Dermatophagoides farinae, Dermatophagoides pteronyssinus, Euroglyphus maynei* as well as storage mites, mostly *Blomia tropicalis* and also *Tyrophagus putrescentiae* and *Lepidoglyphus destructor* are among the main HDM allergen sources in the world [5]. In turn, with the gradual development of molecular allergology it has been established that 30 allergenic molecules of HDMs can cause sensitization in a human [1].

However, the number of elicitors is much bigger. According to http://allergen.org/, the official website of allergens systematic classification, approved by the WHO and the Allergen Nomenclature Sub-committee of the International Union of Immunological Societies (WHO/IUIS), *Dermatophagoides pteronyssinus* (European house dust mite) alone contains 32 allergenic proteins and *Dermatophagoides farinae* (American house dust mite) has 37 such proteins [6]. The discovery of new allergenic molecules of these mites continues [7]. Two more mite species, *Euroglyphus maynei* and *Dermatophagoides microceras*, contain 5 and 2 currently detected molecular allergens respectively.

Such a large (and continuously growing) number of HDM allergenic molecules undoubtedly makes house dust mites an important source of allergy that requires constant research and attention by doctors all over the world. Moreover, allergic sensitization to house dust mites may cause the further development of other allergies, namely food allergies [8], as well as asthma [9].

The fact that the 11th edition of the International Classification of Diseases (ICD 11) contains a separate nosology coded *CA08.02 Allergic rhinitis due to house dust mite* is also an indicator of the clinical significance of allergy to house dust mites. The Classifier describes the nosology as allergic rhinitis triggered by the exposure to house dust mite allergens to which the affected individual has previously been sensitized [10].

Of the currently known molecular components of HDMs, group 1 and 2 allergens (Der p 1, Der f 1, Der f 2 and Der f 2) as well as Der p 23 are considered to be the major sensitization elicitors on the American and the European continents [1, 11]. However, scientists also include *Dermatophagoides pteronyssinus* Der p 5, Der p 7, Der p 21 allergens in the group of most clinically relevant ones [12, 13].

Sensitivity to these as well as to other molecular mite components makes it possible to perform the component-resolved molecular diagnostics of allergy [14]. This allows the clinician to understand the individual sensitization details of a particular patient and, thus, may lead to prescription of a better treatment regimen to patients with an HDM allergy [15].

It must be taken into account that the individual sensitization pattern of a patient is important, as clinical data indicates that sensitization of particular patients to certain allergens is not permanent and has the potential to change with time. [16]. Thus, the initial sensitization of a patient to one or several HDM molecules may subsequently evolve into poly-sensitization [17], which in turn, makes management of these patients more difficult and demands the active supervision at earlier stages of allergic sensitization to the elicitor. Consequently, stratification of patients with an HDM allergy according to their molecular sensitization profiles and molecular monitoring of AIT-induced IgG responses may enhance the success of AIT [12].

In our previous paper [18], we have determined that the population of Ukraine, like most European populations, is most sensitized to group 1 and 2 allergens as well as to Der p 23. However, other allergens, such as Der p 5, Der p 7, Der p 20 and Der p 21 are also relevant for the population of Ukraine. According to our previous data, 8% of Ukrainian patients, sensitized to molecular components of HDMs demonstrated simultaneous sensitization to Der p 1, Der f 1, Der p 2, Der f 2, Der p 21, Der p 23, Der p 5, Der p 7 allergens. However, the process and nature of the gradual development of sensitivity to new molecules, requiring changes into the management protocol of a patient, remain unstudied.

However, since AIT extracts contain only major allergens and form an IgG response mostly to Der p 1 and Der p 2, less often to Der p 23, and to a lesser extent to other allergens [12], the purpose of our study was to establish the pattern of sensitization development in the Ukrainian population depending on age, as well as determine the period when treatment of patients with sensitivity to HDM allergens would be most effective.

## Methods

In order to reach the set goals, we used data from 20,033 people ranging from 0 to 89 years from 17 different regions of Ukraine undergoing the multiplex allergy test Alex^2^ in 2020- 2022. Allergic disease in the anamnesis (allergic rhinitis, atopic dermatitis, bronchial asthma alone or in combination) was the inclusion criterion for the study. Allergists from different regions of Ukraine prescribed Alex^2^ diagnostics after examination of patients. We did not aim to collect detailed data of their symptoms, but rather arranged a study of the sensitization pattern to HDMs among the Ukrainian population with an allergy in the anamnesis. All the patients before the examination signed the informed consent. Among others, it contained a paragraph stating the possible use of impersonalized patient data for scientific purposes. The Ethics Committees that function in every clinic where the data were collected have approved the research.

The multiplex allergy test Alex^2^ components used for the analysis were the sIgE levels to Der f 1, Der f 2, Der p 1, Der p 2, Der p 5, Der p 7, Der p 10, Der p 11, Der p 20, Der p 21 and Der p 23 allergens in individual patients. According to the reference values of Alex^2^ test, 0.31 kU/L was set as the threshold value that indicated sensitization.

To establish the details of sensitization to HDMs at different ages, we determined statistical patterns of sensitivity to this group of elicitors when patients reached the age of 1, 3, 6, 12, 18, 25, 36, 44 and 60 years. Stratification of children’s age (up to 18) was based on the generally accepted classification, which includes the infant period, up to 1 year, toddler 1 to 3 years, preschool child up to 6, primary school age (6 to 12 years), high school age or puberty (12 to 18 years). The selected ages of adults corresponded with the beginning and ending of youth (25 and 44 years respectively) and the end of middle age (60 years) [19]. Additionally, the age of 36 years was selected as the point between the periods of first and second youth.

To determine the general characteristics of the sample data, namely, the distribution of patients by age, by the level of total IgE and specific sIgE to the individual molecular components of house dust mites, by the level of specific sensitization to these components in separate age groups, the descriptive statistics function of MS Excel 2013 was used.

To select the data of sensitive patients from the general sample we used a specially developed algorithm. By sorting the patients’ data, this algorithm deleted those, who did not meet the sensitivity level at which the data were used for calculations. The sensitivity level used to conduct statistical calculations was 0.31 kU/L as a referent for the Alex^2^ test. If the values were lower, the data were not considered.

To determine the probability of developing sensitization to certain molecular components in individual patient profiles, machine learning was used as it has been previously shown to have applications in the diagnostics of allergic diseases, in particular for children [20]. Thus, according to data on sensitization to HDM allergens in the study population, a Bayesian network was built, as a tool to determine the probability of developing processes related to the health of the population [21] useful for analyzing the data obtained from the eHealth system [22].

A Bayesian network (BN) is a directed acyclic graph (DAG). A directed graph is acyclic if it has no paths that begin and end at the same node. In addition, a graph is directed if the nodes are linked by directed edges, which show how one edge influences the other. Each DAG built with the given tabular data allows us to draw certain conclusions about the probability of some events, depending on the probability of other events. As a rule, the methods of machine learning with BN application are narrowed down to the search for one of three types of patterns [21]:

– Causal chain:

1 $$X\to Y\to Z$$

– Common effect:

2 $$X\leftarrow Y\to Z$$

– Common cause:

3 $$X\to Y\leftarrow Z$$

where X, Y, Z are some variables (e.g. presence of sensitization to a certain allergen), but X and Z are independent variables, which are being analyzed under certain conditions regarding Y. That is, if one of the three BN types (1)–(3) is found in the table of data with three characteristics X, Y, Z, certain conclusions can be drawn about the probability of having one characteristic in a given object, depending on the probability of having other characteristics.

This approach is usually used for finding causative-consecutive patterns [21–25]. Nevertheless, we suggest using it in a broader way. In fact, the above equations lack the explicit time coordinate. There is usually just a dataset about N objects with M characteristics. A comparison of hypotheses, regarding the way the probabilities of compatible events are compared, is performed. For instance, a group of patients have characteristics X = “1” and Z = “0” if characteristic Y = “1”. Then we may conclude that there is a type (2) pattern. In real conditions, for every “1” or “0” value of X, Y, Z characteristics, there is a probability table of these values and then condition (2) acquires its probabilistic nature. There are special methods of condition (1)–(3) identification within datasets. The main result of the established patterns (1)–(3) is the probability of the presence of one characteristic, which may or may not appear depending on other characteristics. However, the important part is that due to the mathematical formalization of the task, we receive quantitative characteristics of the probabilities that may be compared. For example, for Y = 0 there is a ${p}_{1}$ probability of a certain event; and for Y = 1 it is 3 ${p}_{1}$. Then, we can make a mathematically grounded conclusion that characteristic value Y = 1, triples the probability of the event’s occurrence.

For instance, analyzing the dataset, we can construct the patterns of probability distribution for the values of presence (value “1”) and absence (“0”) of patients’ sensitivity to allergens, conditionally, D1, D2 and D3 and others, for which data are available, either for each elicitor separately, or together in all possible combinations. Then the analysis may give the following results: sensitization to D1, in the absence of sensitivity to D2, increases the probability of sensitization to D3 up to 90%. Instead, the lack of sensitivity to D1 and D2 determines the probability of developing sensitivity to D3 with a probability of 60%. If there is a sensitivity to D2, the probability of sensitization to D3 is reduced to 45%. It should be noted that this is not a causative-consecutive connection in time, but rather a classical set of events, when events are considered in a comprehensive manner and the presence or absence of some events is analyzed, in the presence or absence of other events, with this or that probability. In the case of sensitization to individual molecules of HDMs, it is possible to determine the patterns of combined sensitivity to them in the individual profiles of the patients.

In our case, the DAG root node was set to Der p 23, and with the help of bnlearn.bnlearn.parameter_learning.fit function, we calculated the values of conditional probability distribution (CPD) of connections between separate allergens in the individual patient profiles for each of the studied molecules.

## Results

### Characteristics of the patients

**Fig. 1:**
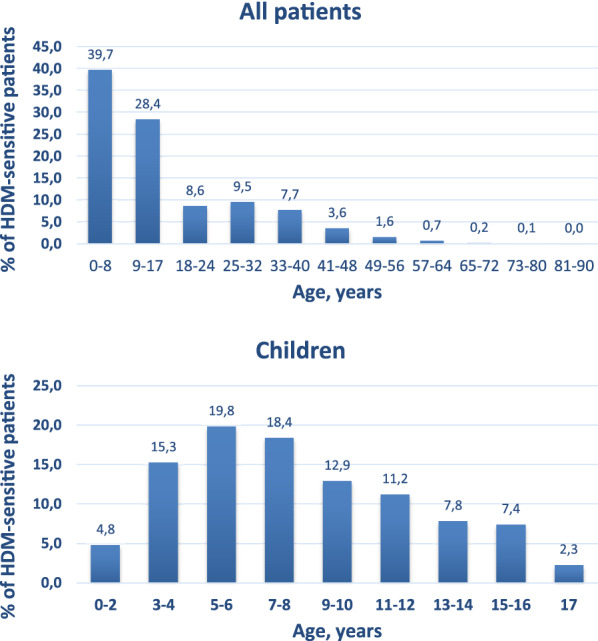
Age range of the studied subjects sensitive to house dust mites

Sensitization to at least one HDM allergen was detected in 5170 people, which made up 25.81% of the total study population. The number of sensitized children under 18 was 2.13 times higher than the number of adults with 3518 (68.05%) children vs 1652 (31.95%) adults. The mean age of the HDM-sensitized group was 15.87 years. The median age was 11 years. The mean age for children was 8.20 years and 32.19 years for adults. The group of 0–8-year-old children was the largest in the sample. In the children’s group, 5–6- and 7–8-year-old children prevailed. Among adults, the age groups 18–24 and 25–32 years prevailed (Fig. 1).

### General patterns of sensitization to HDM allergens in the study sample

Patients that were included in the study had prevailing sensitization to Group 2 allergens. Sensitivity to Group 2 allergens, either mono-sensitivity or combined with sensitivity to other allergens, was observed in practically the same number of patients (72.40% were sensitized to Der f 2 and 71.59%—to Der p 2); 54.99%, 54.99% and 52.61% people were sensitive to Der p 23, Der f 1 and Der p 1 respectively. These allergens were followed by Der p 5, Der p 21 and Der p 7, Der p 20, Der p 10 and Der p 11.

The highest registered sIgE level in children as well as in adults was to Der f 2 and Der p 2 allergens. A similar situation could be observed in children and adult groups separately. In the children group, the mean sIgE level was 32.31 ± 16.76 kU/L to Der f 2 and 31.69 ± 16.90 kU/L to Der p 2. In the adult group, the mean sIgE level was 19.16 ± 16.32 kU/L to Der f 2 and 18.07 ± 15.95 kU/L to Der p 2. The third place was occupied by Der p 21 (20.26 ± 17.49 kU/L for the general sample with 24.65 ± 17.57 kU/L for children and 11.37 ± 13.50 kU/L for adults). The lowest registered sIgE level was to Der p 11. Next to last were Der p 20 for the whole sample and for children, as well as Der p 23 for the adult group. As we can see, sIgE levels to practically all allergenic components were considerably lower in older groups. In most cases they were half or almost half lower in adults. The exceptions were sIgE levels to Der p 10, Der p 11 and Der p 20, which were of almost the same level in different age groups, and in case of Der p 11 the index even slightly increased in the adults (Table 1). The mean tIgE level for the whole HDM-sensitized group was 324.12 kU/L. The mean tIgE level in the children group was 371.23 kU/L, the mean tIgE level in the adult group was 222.51 kU/L.

**Table 1:** Number, share and average sIgE values of patients, sensitized to molecular allergens of house dust mites

HDM molecules	slgE value, kU/L	Number of patients sensitive to the allergen	Share of patients sensitive to a respective allergen from the whole sample of patients, %	sIgE value in the children group, kU/L	Number of children sensitive to a respective allergen	Share of children sensitive to a respective allergen, %	sIgE value in the adult group, kU/L	Number of adults sensitive to a respective allergen	Share of adults sensitive to a respective allergen, %
Der f 1	16.16 ± 14.77	2821	54.56	19.29 ± 15.10	2083	40.29	7.30 ± 9.21	738	14.27
Der f 2	28.18 ± 17.71	3743	72.40	32.31 ± 16.76	2568	49.67	19.16 ± 16.32	1175	22.73
Der p 1	14.19 ± 13.20	2720	52.61	16.71 ± 13.57	1981	38.32	7.43 ± 9.20	741	14.33
Der p 10	12.48 ± 15.99	320	6.19	13.34 ± 16.47	207	4.00	10.91 ± 14.95	114	2.21
Der p 11	1.26 ± 2.72	30	0.58	0.94 ± 1.17	14	0.27	1.53 ± 3.54	16	0.31
Der p 2	27.45 ± 17.77	3701	71.59	31.69 ± 16.90	2548	49.28	18.07 ± 15.95	1153	22.30
Der p 20	9.73 ± 13.10	438	8.47	11.49 ± 14.56	234	4.53	7.72 ± 10.85	204	3.95
Der p 21	20.26 ± 17.49	1422	27.50	24.65 ± 17.57	952	18.41	11.37 ± 13.50	470	9.09
Der p 23	15.77 ± 15.31	2843	54.99	19.47 ± 15.66	2011	38.90	6.81 ± 9.72	832	16.09
Der p 5	16.00 ± 15.35	1512	29.25	19.56 ± 15.72	1065	20.60	7.54 ± 10.32	447	8.65
Der p 7	17.07 ± 15.79	1172	22.67	20.37 ± 16.00	834	16.13	8.92 ± 11.80	338	6.54

### Determining sensitivity to HDM allergens depending on the age of patients

The number of patients in the stated age groups is significant and thus sufficient for obtaining viable sensitivity patterns for each group. The results of the studying the change in the nature if sensitivity to HDMs allergens in different age groups have shown that the main sensitization pattern is much the same in all age groups. Namely, by sensitization to HDM allergens of groups 1 and 2 and also to Der p 23 becomes pronounced by the time a person reaches 1 year of age. However, in older people the increased sensitivity to group 2 allergens and a relative decrease in the sensitivity to group 1 allergens is observed whereas the sensitivity incidence to Der p 23 allergen remains significant in all age groups. This is the only leading elicitor the incidence in sensitivity to which does not undergo significant changes and remains significant even at the age of 60.

**Fig. 2:**
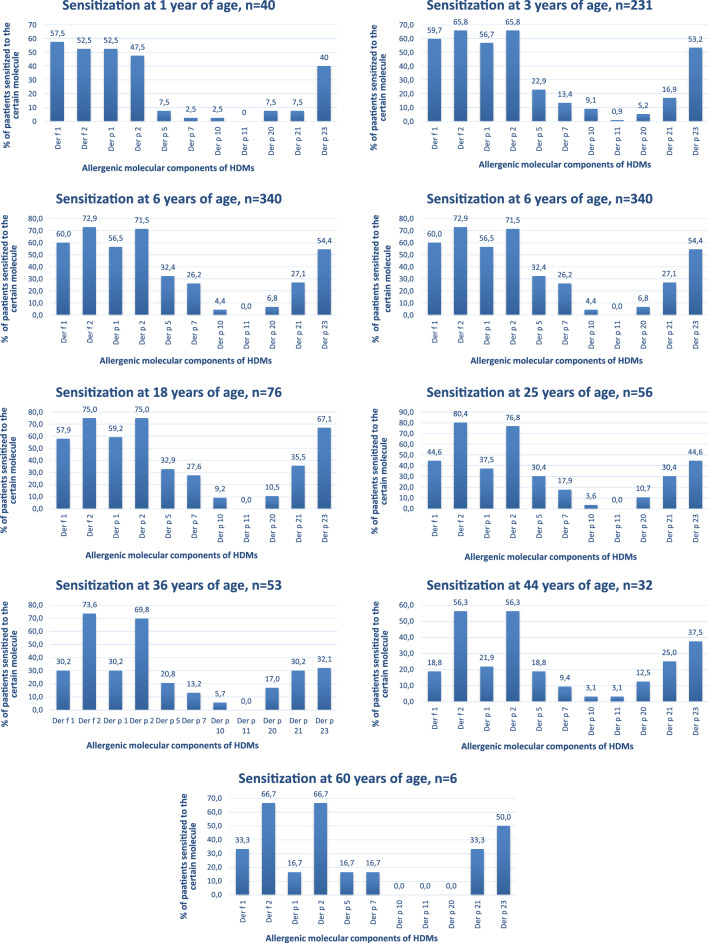
Specifics of sensitization to house dust mites among patients of different age groups in Ukraine

A considerable incidence of sensitivity was also observed to Der p 5, Der p 7 and Der p 21 allergens. However, its significance becomes more pronounced starting from the age of 3–6 years. The lowest incidence in all age groups was observed to Der p 10, Der p 11 and Der p 20 allergens. (Fig. 2).

### The analysis of probabilistic connections between sensitization to different HDM molecular components using Bayesian network analysis

**Fig. 3:**
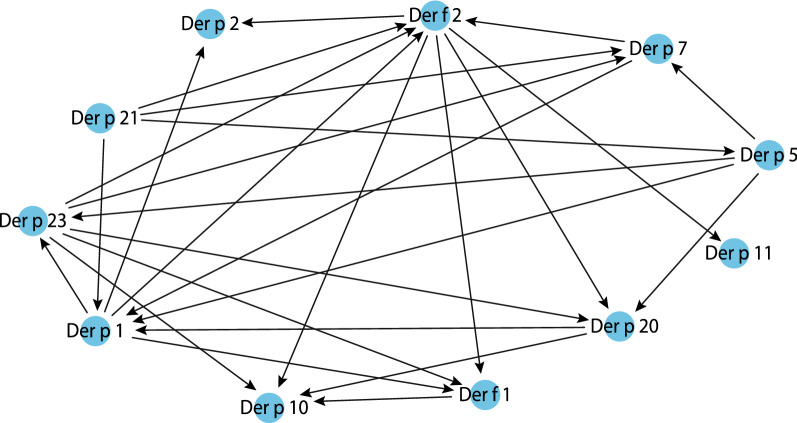
The resulting Bayesian directed acyclic graph of probabilistic connections between individual allergenic molecules in the individual patient profiles of the studied sample

The age changes in sensitization described above are characteristic of certain age groups not of individual patients. To assess the patterns of individual sensitivity to HDM allergens among the Ukrainian population, we used Bayesian network analysis with its root node set to Der p 23 (Fig. 3). This allergen was selected because the sensitivity to Der p 23 remained considerably high in all age groups.

Conditional probability distributions (CPD) at which the patient profile demonstrated sensitivity to the root node Der p 23 with the simultaneous sensitization to Der p 1, Der p 5 and Der p 7 reached 82.17%. The CPD of the absence of sensitivity to all these allergens was 63.14%. Der f 1 allergen experienced the highest probabilistic influence of Der f 2, Der p 1, Der p 23 molecules. The CPD of presence of all 4 molecules in the profile was observed in 93.62% of the patients.

Der p 10 was connected with Der f 1, Der f 2, Der p 20 and Der p 23 allergens. However, the CPD of presence of all these molecules in a patient’s profile was only 31.57%. At the same time, the CPD of sensitization to Der f 1, Der f 2, Der p 20 and Der p 23 increased more than twice—up to 68.43%—if Der p 10 was absent from the profile. In addition, CPD of absence for all these allergens was also significant at 59.87%.

The presence of Der f 2 allergen was most probabilistically connected with Der p 1, Der p 21, Der p 23 and Der p 7 molecules. The CPD of all of the above-mentioned molecules was 91.25%. However, even with the absence of sensitization to all of the named molecules, the probability of sensitivity to Der f 2 alone was high and reached 62.60%. Another major allergen, Der p 2, had mostly probabilistic connections with Der f 2 as well as with Der p 1. The probability of the presence of all three molecules in a profile was 93.88%. Der f 2 was the only allergen, probabilistically connected with Der p 11. But both allergens together were found in only 7.46% of patients. In contrary, the CPD of Der f 2 presence at the absence of Der p 11 was 92.54%.

The presence of the Der p 20 allergen in the profile was influenced by sensitization to the Der f 2, Der p 1, Der p 23 and Der p 5. If there was no sensitization to all of these molecules, there was a 35.94% probability of sensitization to Der p 20.

**Fig. 4:**
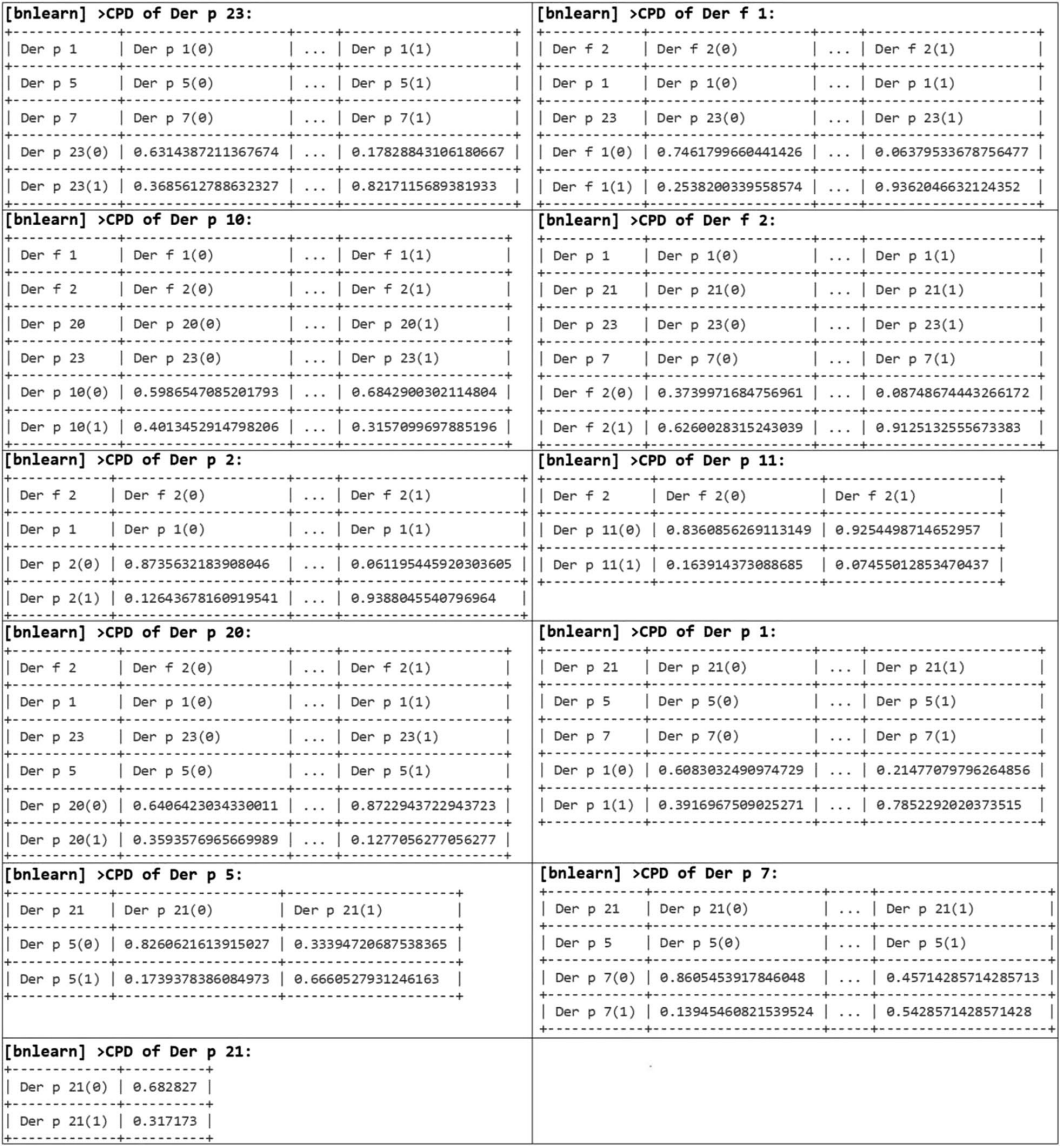
Conditional probability distributions of house dust mites’ molecules in Bayesian network

Der p 1 was most influenced by Der p 21, Der p 5, Der p 7 molecules. The CPD of sensitization to all 4 molecules was 78.52%. The CPD of absence of all of the stated molecules in a profile was 60.83%. The Der p 21 allergen was probabilistically connected with Der p 5 and Der p 7 allergens but was not probabilistically affected by other allergens. The estimated probability of independent sensitivity to Der p 21 was 31.72%. When there was sensitivity to Der p 21, the probability of sensitivity to Der p 5 was 66.1%. At the same time, 82.61% of patients did not demonstrate probabilistic sensitivity to either of these allergens. With the absence of sensitivity to Der p 21 and Der p 5, and the sensitivity to Der p 7 also absent, the probability was 86.05%. (Fig. 4).

## Discussion

Using the Bayesian network method, it was possible to conduct a detailed analysis of individual sensitization profiles of patients. Moreover, the described sensitization to a greater number of mite allergens in patients of older age may be of great clinical significance. However, very little data are currently available on the possibility of comparing different molecular sensitization patterns with clinical phenotypes [15].

It is known that specific therapeutic approaches do not yet take into account the local sensitization profiles of patients [1]. The results of our research show that the general character of sensitization to HDM allergens are similar in all age groups. Ukrainians were most often sensitized to HDM allergens of groups 1 and 2, as well as to Der p 23. This is consistent with the data of other authors on the sensitization of the population in Europe [13, 26]. However, this is in contrast to the African continent, where group 1 and Der p 23 allergens were also important, and the major allergen was Der p 7 with 56% of sensitized patients [1]. The latter remained minor in Ukraine, which indicates the importance of studying regional sensitization specifics in order to choose the right algorithm for allergen immunotherapy (AIT).

The tendency that could be observed in Ukraine is that the percent of patients sensitized to group 2 allergens is lower at younger age. Sensitivity of a high percent of younger children to group 1 allergens could, among other things, be explained by the fact that Der p1 allergen was found in breast milk [27].

Der p 5, Der p 7 and Der p 21 allergens were relatively important in all age groups except children under one, while sensitization to Der p 10, Der p 11, Der p 20 was least significant in all age groups. This confirms the statements expressed by other authors, who claim that sIgE levels were little influenced by age, gender or allergen type [28].

The analysis with the use of Bayesian network has confirmed the fact that the most probable sensitivity in individual patient profiles is the simultaneous sensitivity to allergens of groups 1 and 2, as well as to Der p 23. The major significance of sensitization to group 1 and 2 allergens as well as to Der p 23 in the study population is consistent with the data of other authors [29].

Der p 1 and Der f 2 were determined as the leading molecules in groups 1 and 2 respectively. Each of them, according to DAG, probabilistically determined the presence of five other allergenic molecules (from their own group as well as from other groups) in the profiles of sensitized patients.

Der p 21, Der p 23 and Der p 5 probably each determined the presence of 4 other allergenic molecules in a profile, which also indicates their practical significance and aligns with the data published by Weghofer et al. [30] and Pang et al. [31]. Namely, these authors have demonstrated the homology of Der p 5 and Der p 21 allergens, which both belong to group 5 of HDM allergens. And Kim et. al. [32] determined a 36.6–55.8% of sequence identity of Der p 5 and Der p 21 allergens. The data we obtained may indicate both significant probability of cross-reactivity between these two allergens – against the background of sensitivity to Der p 21, the probability of sensitivity to Der p 5 was 66.61% – as well as the leading role of Der p 21 in this pair. The DAG showed that actually sensitization to Der p 21 probabilistically determines the development of sensitivity to Der p 5. This hypothesis requires further practical study.

An interesting fact is that the same Der p 21, together with Der p 1, Der p 23 and Der p 7, was conditionally connected with the presence of the Der f 2 allergen in individual patient profiles. In turn, Der p 23 was connected with Der p 1, Der p 5 and Der p 7.

As we can see, in addition to the already mentioned group 1 and 2 allergens and Der p 23, Der p 21, Der p 5 associated with Der p 21, as well as Der p 7 are the ones most probably present in individual patient profiles. The connection of the latter with the previous ones was described by Lynch N.R et al. [33], who revealed the existence of a significant immunological relationship between Der p 5 and Der p 7. This connection was also confirmed by calculations performed using the Bayesian network: in the absence of sensitivity to Der p 21 and Der p 5 in 86.05% of patients there was no sensitivity to Der p 7. The allergenic activity of Der p 7, comparable to that of Der p 5, Der p 21 and Der p 23, was also indicated by Mirela Curin et al. [34].

Analysis of research on the clinical significance of mite allergens, attempting to determine the most important ones for the Ukrainian sample, revealed that early sensitization in children under 5 to Der p 1, Der p 2 and Der p 23 allergens was associated with the development of bronchial asthma in school-age children [16]. Sensitization to Der p 23 should be taken into account in the diagnosis and treatment of mite allergy, especially in patients with a moderately severe asthma for such sensitization can cause a more severe course of the clinical phenotype. [35, 36]

According to the data presented in scientific papers, Der p 7 is associated with the development of allergic rhinitis and sensitization to Der f 2, Der p 2 and Der p 23 increases the risk of developing atopic dermatitis. At the same time, sensitization to Der p 10 reduces the risk of atopic dermatitis development [29]. Our data indicate that such effect may be connected with the absence of sensitization to group 1 and 2 allergens as well as to Der p 23 in patients whose profiles contain Der p 10 as CPD of sensitization to Der f 1, Der f 2, Der p 20 and Der p 23 increased up to 68.43% if Der p 10 was absent in the profile (Fig. 4).

AIT remains the main management strategy for HDM sensitized patients. It can relieve AR symptoms [37] and also improve the lung function of children and teenagers with the symptoms of HDM caused asthma [38]. Individual profiles of sensitization to HDM allergens can vary greatly and such heterogeneity should be taken into consideration while diagnosing and treating patients with HDM allergy [39]; it has been proven that AIT induces the proliferation of IgG and IgG4, specific for HDM allergens Der p 1, Der p 2 and Der p 23 and, to a lesser extent, other allergens. However, scientists [34, 36] also indicate the potential of creating products for AIT to work with Der p 5 and Der p 7 allergens. The efficacy of AIT in patients with sensitization to Der p 5, Der p 7, Der p 20, Der p 21 allergens is actually the subject for further research, and the selection of house dust mite-allergic patients by molecular diagnosis may enhance the success of AIT [40] and improve the clinical description of patients [41].

Another controversial issue, which is also a subject for further research, is the minimum age for prescription of AIT. We have shown that for many patients sensitization profiles to HDMs are formed during the first year of life.

## Conclusions

Thus, the data indicate that general character of sensitization to HDM allergens is similar in younger and older patients and residents of Ukraine are most often sensitized to HDM allergens of group 1 and 2 as well as to Der p 23. A considerable sensitivity incidence to Der p 5, Der p 7 and Der p 21 was also observed. Both in children and adults, the highest sIgE levels were registered to Der f 2, Der p 2 and Der p 21 allergens.

Results of the Bayesian Network analysis of personal profiles indicated the leading role of Der p 1 and Der f 2 in the 1st and 2nd groups respectively and that the leading role in the formation of sensitivity to group 5 allergens may belong to Der p 21.

These data show the importance of determining the sensitization profile using the multi-component approach, which tests the highest number of mite molecules, in order to apply a targeted treatment of an HDM allergy.

## Data Availability

Principles of Bayesian Network construction and their results for the given dataset can be seen at https://www.kaggle.com/code/vbmokin/der-bayesian-network-analysis/. Primary patients’ data can be shared particularly just following email request to the corresponding author due to potential sensitivity of the data of individual patients.

## Abbreviations

AIT: Allergen immunotherapy
AR: Allergic rhinitis
CPD: Conditional probability distribution
HDM(s): House dust mite(s)
ICD: International classification of diseases
IUIS: International Union of Immunological Societies
WHO: World Health Organization
